# Risk of non-melanoma skin cancer in patients with psoriasis: An updated evidence from systematic review with meta-analysis

**DOI:** 10.7150/jca.37015

**Published:** 2020-01-01

**Authors:** Xiujuan Wang, Qiang Liu, Lingling Wu, Zhenhua Nie, Zubing Mei

**Affiliations:** 1 Department of Dermatology, Tianjin Academy of Traditional Chinese Medicine Affiliated Hospital, Tianjin, People's Republic of China.; 2 Department of Medical Acupuncture, The First Affiliated Hospital of Tianjin University of Traditional Chinese Medicine, Tianjin, People's Republic of China.; 3 National Clinical Research Center for Traditional Chinese Medicine, Tianjin, People's Republic of China.; 4 Department of Anorectal Surgery, Shuguang Hospital, Shanghai University of Traditional Chinese Medicine, Shanghai, China.; 5 Anorectal Disease Institute of Shuguang Hospital, Shanghai, China.

**Keywords:** Non-melanoma skin cancer (NMSC), Psoriasis, Risk ratio, observational study, Meta-analysis.

## Abstract

***Background***Psoriasis is a chronic inflammatory skin disorder which may result in an increased cancer risk due to defects of immune surveillance. The relationship between psoriasis and risk of non-melanoma skin cancer (NMSC) has not yet been fully determined. The aim of this study was to update the evidence on the association between psoriasis and risk of NMSC.

***Methods***We conducted an extensive literature search of publications in Pubmed, EMBASE, and Cochrane Library without restrictions on language from inception through August 2019 using predefined keywords. Eligible observational studies were selected if they assessed the risk ratio of NMSC in patients with psoriasis. Data from included studies were extracted, and meta-analysis was performed using random-effects models.

***Results***Sixteen cohort studies involving 16,023,503 participants published between 1999 and 2019 met inclusion criteria and were included in this systematic review. Meta-analysis demonstrated that compared with patients without psoriasis, patients with psoriasis had 1.72 times higher risk of developing NMSC (RR, 1.72, 95% CI 1.46 to 2.02). Patients with moderate to severe psoriasis had higher risk of NMSC (RR, 1.82, 95% CI 1.38 to 2.41) than those had mild psoriasis (RR, 1.61, 95% CI 1.25 to 2.09) (P for interaction<0.001). Moreover, patients with psoriasis had significantly higher risk of squamous cell carcinoma (RR, 2.08, 95% CI 1.53 to 2.83) than that of basal cell carcinoma (RR, 1.28, 95% CI 0.81 to 2.00) (P for interaction<0.001).

***Conclusions***Current evidence suggests that patients with psoriasis may have a higher risk of NMSC than psoriasis-free patients. Periodic screening for specific cancer risk is warranted in patients with psoriasis.

## Introduction

Psoriasis is a chronic inflammatory and immune disease that affects more than 2-3% of the world's general population.^1^, ^2^ Psoriasis mainly damages skin with the main pathological features of excessive growth and differentiation of keratinocytes. It has been reported that the immune cells, especially T cells were involved in the immune response, which has a major impact on the pathogenesis of psoriasis.^3^ Studies also show that psoriasis shares several clinical features with other chronic illness including psoriatic arthritis, metabolic syndrome, depression, cardiovascular diseases and even malignancies. 4-6

Due to the exposure to immunosuppressive agents, methotrexate, cyclosporine, and UV therapies, there seems to be increased cancer risk in patients with psoriasis.^7^, ^8^ However, the association between psoriasis and the risk of non-melanoma skin cancer (NMSC) is still controversial. Several epidemiological studies have demonstrated an increased risk of skin cancers in Caucasian patients with psoriasis who had psoralen plus ultraviolet A (PUVA) therapy.^9^-^12^ However, several other cohorts found the opposite results.^13^, ^14^ Thus, it is currently uncertain whether psoriasis is associated with elevated risk of NMSC. Given the accumulating epidemiological evidence associated with patients with psoriasis, it is considered clinically important to gain a better understanding if psoriasis condition really affects NMSC risk.

We therefore performed a systematic review and meta-analysis of observational studies investigating the association between psoriasis status and risk of NMSC. The primary aim of this systematic review and meta-analysis was to precisely gauge the nature and magnitude of this association in patients with psoriasis. We also assessed whether the severity of psoriasis can have an impact on the development of NMSC and whether psoriasis status can affect different pathologic type of NMSC.

## Methods

### Literature search

A systematic review was performed based on the Preferred Reporting Items for Systematic Reviews and Meta-Analyses (PRISMA) guidelines.^15^ We also followed the Meta-analysis Of Observational Studies in Epidemiology (MOOSE) guidelines for the meta-analysis of these studies,^16^ in that we involved observational studies.

We conducted an extensive literature search of publications in Pubmed, EMBASE, and Cochrane Library without restrictions on language from inception through August 2019 for identifying observational studies examining the association between psoriasis and risk of NMSC. The search free text terms were “psoriasis” AND (“skin neoplasms” OR “non-melanoma skin cancer” OR “NMSC” OR “keratinocyte carcinoma” OR “basal cell carcinoma” OR “squamous cell carcinoma”). MeSH (Medical Subject Headings) terms were also searched (**Supplementary Search Strategy**). We did not apply language restrictions. In addition, manual search of potentially additional citations within references of the included studies, reviews and meta-analyses was also carried out for locating additional suitable studies not found by the electronic database searches. A search for unpublished literature was not applied.

### Study selection

The study inclusion criteria included (i) being population-based or hospital-based prospective or retrospective study with cohort, case-cohort or nested case-control design; report adjusted estimates of the relative risk (RR) [e.g. hazard ratio (HR), risk ratio or odds ratio (OR)] with corresponding 95% confidence intervals (CIs) for the association of psoriasis and NMSC risk. When the same cohort publishedmore than one article on psoriasis and NMSC risk, the most comprehensive one with the largest sample size was selected.

### Data extraction and quality assessment

Two reviewers (XW and QL) independently selected studies that potentially satisfied the inclusion criteria based on their titles or/and abstracts. If necessary, we would retrieve the full text articles for a more detailed assessment and eligibility for inclusion. For each included study, two reviewers (XW and QL) independently extracted related data including first author, year of publication, study country and design, study setting, cohort sample size, percent of female, measure of psoriasis, outcome ascertainment, mean/median follow-up period, adjusted covariates, and OR, HR, RR, SIR along with their associated 95% CIs. The results of the abstracted data were cross-checked. Any disagreements were resolved by discussion or contacted a senior reviewer (ZM).

The Newcastle-Ottawa scale (NOS) was used to assess study quality for observational study. This scale, ranging from 0 to 9 scores assigned for participant selection, comparability and outcome (cohort) or exposure ascertainment (case-control), and the potential for confounding.^17^ We defined a total NOS score being 7 or more as high quality study^49^ and a score being less than 7 as low quality study.

### Statistical analysis

Data analysis was undertaken using Stata® version 13.0 (Stata Corp LP, College Station, Texas, USA). A random-effects meta-analysis was performed to calculate the summary RRs and the 95% CIs, using the approach described by DerSimonian and Laird et.al.^18^ Inter-study heterogeneity was examined using I^2^ statistic defining the percentage of the total variation across studies, with an I^2^ more than 50% representing significant heterogeneity.^19^ To examine the potential sources of heterogeneity, subgroup analyses were conducted by geographic continent, study setting, sex, sample size, follow-up period, study quality, adjustment for major variables, psoriasis severity and pathologic type. Besides, visual inspection of funnel plot asymmetry combined with Egger's regression^20^ and Begg's rank correlation tests^21^ was used to test publication bias with a P< 0.05 indicating the presence of publication bias. Finally, trim-and-fill approach was also applied to explore the potential influence of publication bias by calculating the number of missing studies that might exist in a meta-analysis. 22 A cumulative meta-analysis by sorting of the included studies was also conducted based on the date of study publication.

## Results

### Literature Search and Study characteristics

**Figure 1:**
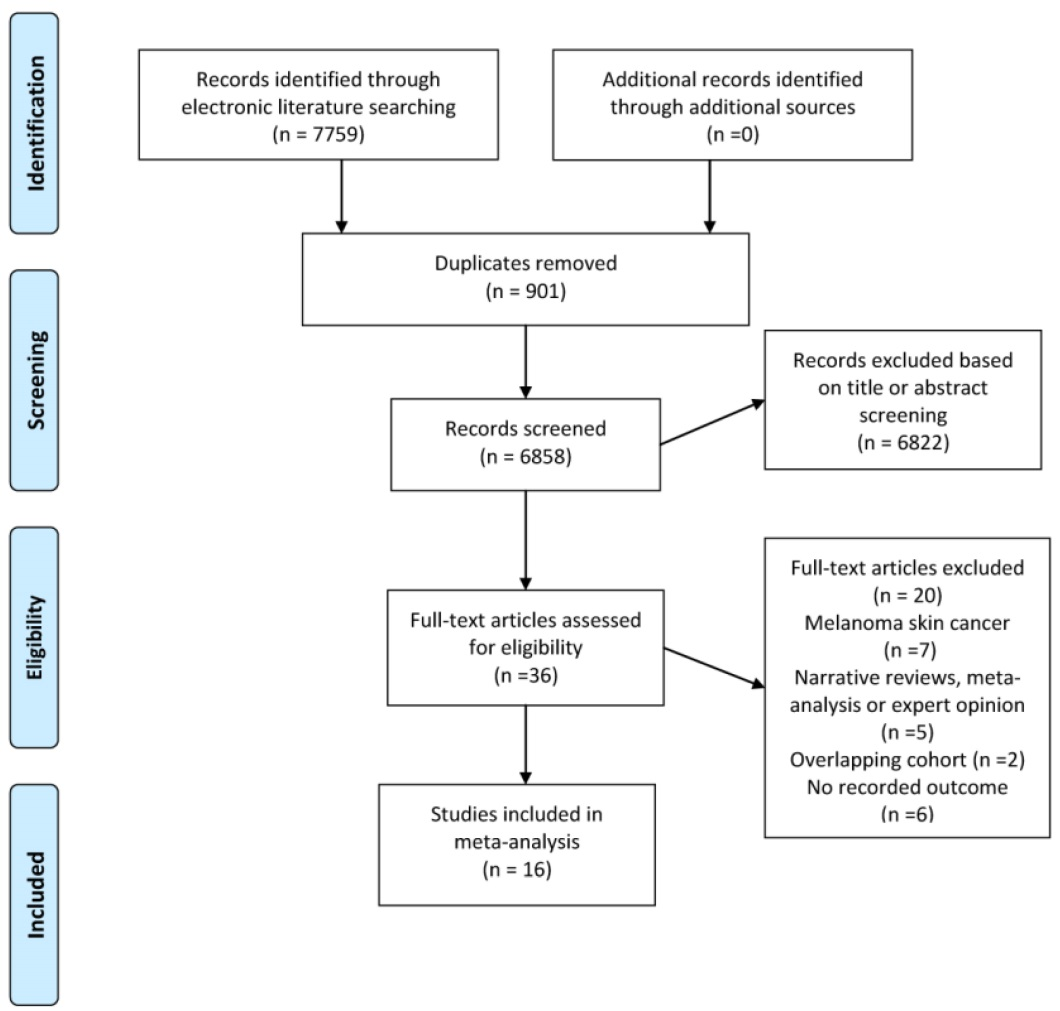
Flow chart of the evidence search and study selection process.

**Figure 1** demonstrates the flow diagram for literature selection and inclusion in this meta-analysis, which generated 7759 records. Thirty-six potentially relevant citations were then identified for full text review. Finally, a total of 16 cohort studies involving 16,023,503 participants published between 1999 and 2019 met the inclusion criteria and were included in the final analysis.^13^, ^23^-^35^, ^50^, ^51^ The median sample size was 173,170 (range, 5687 to 5559420). The median follow-up period ranged from 1 to 16 years. We presented the summary descriptions of each included study in **Table 1.** In summary, all studies were published between 1999 and 2019, with 11 studies involved population-based cohorts, and 5 had hospital-based cohorts. Six of the studies examined populations from North America, seven studies were from European countries and three investigated Asian populations. Eight studies used regression model adjusted for major variables such as sex, age and body mass index. In terms of method for ascertainment of psoriasis and NMSC, most of the studies used a diagnosis based on International Classification of Diseases (ICD) criteria collected in the database.

### Meta-analysis

**Figure 2:**
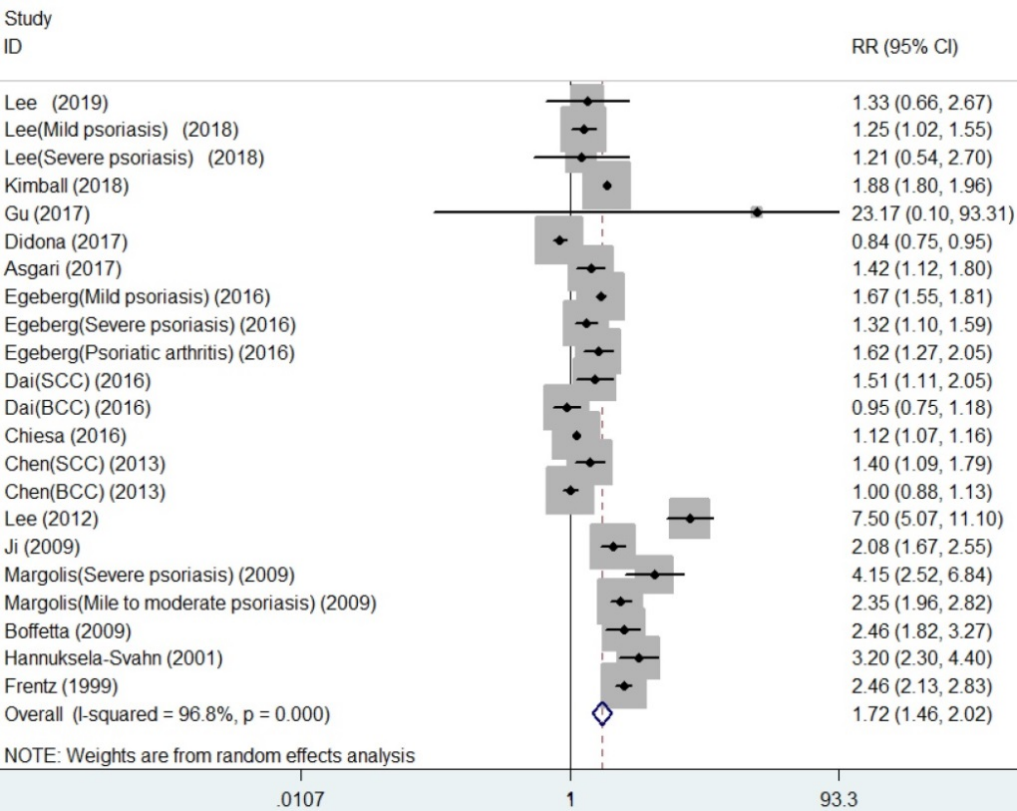
Forest plot for association between psoriasis and risk of non-melanoma skin cancer.

As shown in **Figure 2**, the summarized data from the included 16 studies indicated that compared with patients without psoriasis, patients with psoriasis had 1.72 times higher risk of developing NMSC (RR, 1.72, 95% CI 1.46 to 2.02), with significant heterogeneity across studies (I^2^=96.8%).

**Table 2** gives the results of detailed subgroup analyses by potential sources of heterogeneity among some of the major clinical features of the included studies. The pooled RRs for the majority of subgroups remained constant by the study features, including geographic continent, study setting, sample size, follow-up period, adjusted variables, study quality, and psoriasis severity. A possible interaction was noted in four features (geographic continent, follow-up period, psoriasis severity and pathologic type), although we had conducted multiple hypothesis testing by nine features. Though we did not observe significant associations between psoriasis and risk of NMSC for male patients (RR, 1.99, 95% CI 0.73 to 5.43) or for basal cell carcinoma (RR, 1.28, 95% CI 0.81 to 2.00), these associations should be further examined due to the small number of studies in each of the subgroup.

### Study quality, publication bias and sensitivity analysis

The methodological quality score of the included studies was moderate to high in 88% (14/16) of the included studies based on the NOS score (**Supplementary Table 1**); most studies used population-representative subjects, had clearly reported the ascertainment of exposure, adequate length of follow-up, had a sufficient measurement of outcomes, and only one study with abstract form (27) rated low quality for lack of information. However, subgroup analysis based on the study quality yielded similar results between high quality studies (RR, 1.56, 95% CI 1.28 to 1.91) and low to moderate studies(RR, 1.99, 95% CI 1.39 to 2.84) (P=0.805). We also conducted sensitivity analysis by omitting the low quality study and recalculating the others, which did not alter the result largely.

**Figure 3:**
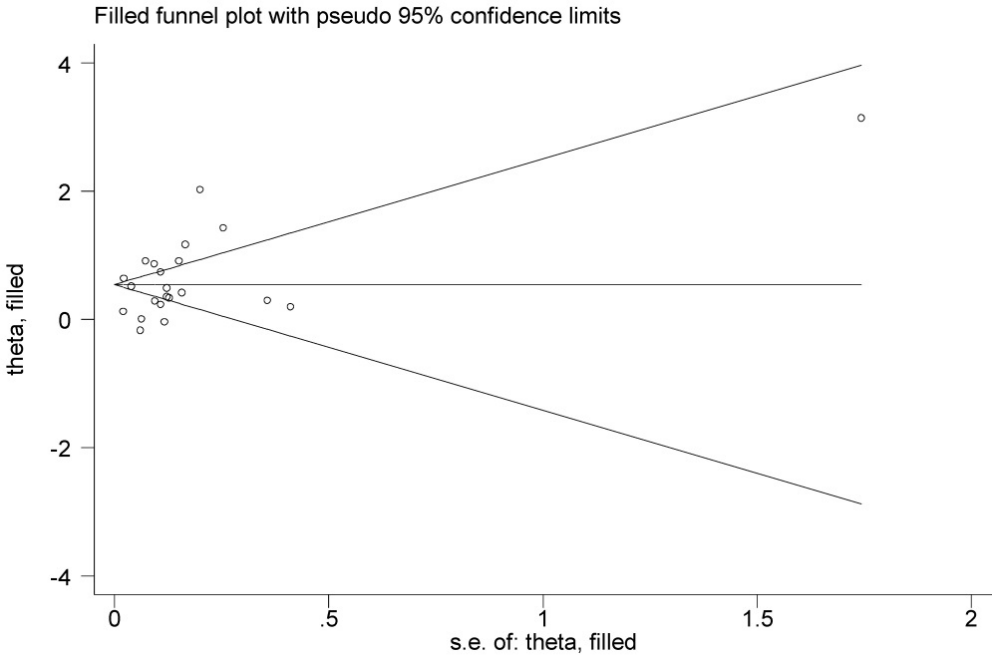
Filled funnel plot for association between psoriasis and risk of non-melanoma skin cancer.

Publication bias analysis indicated a little asymmetry of the funnel plot. However, Begg's rank correlation test (P=0.398) or Egger's test (P=0.324) did not suggest the asymmetry was attributed to publication bias. Thus, we used the Duval's nonparametric trim-and-fill method to adjust the pooled RR, and the result was the same with that of the primary analysis, showing the robustness of the result (**Figure 3**).

**Figure 4:**
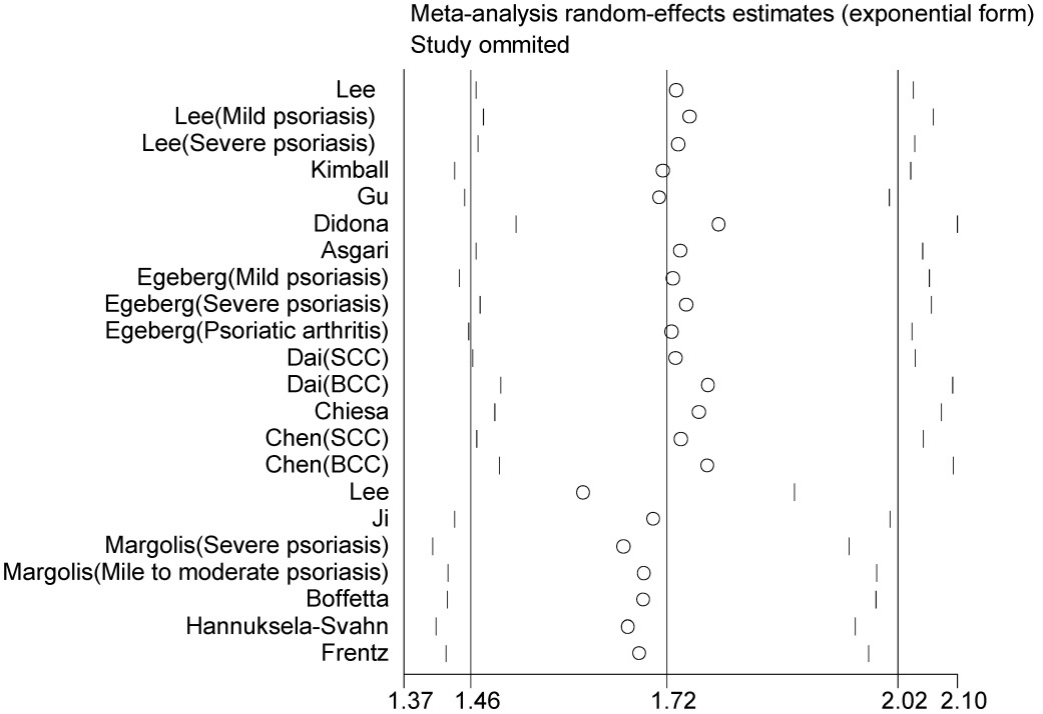
Sensitivity analysis by omitting individual study at one time on the summary relative risk.

**Figure 5:**
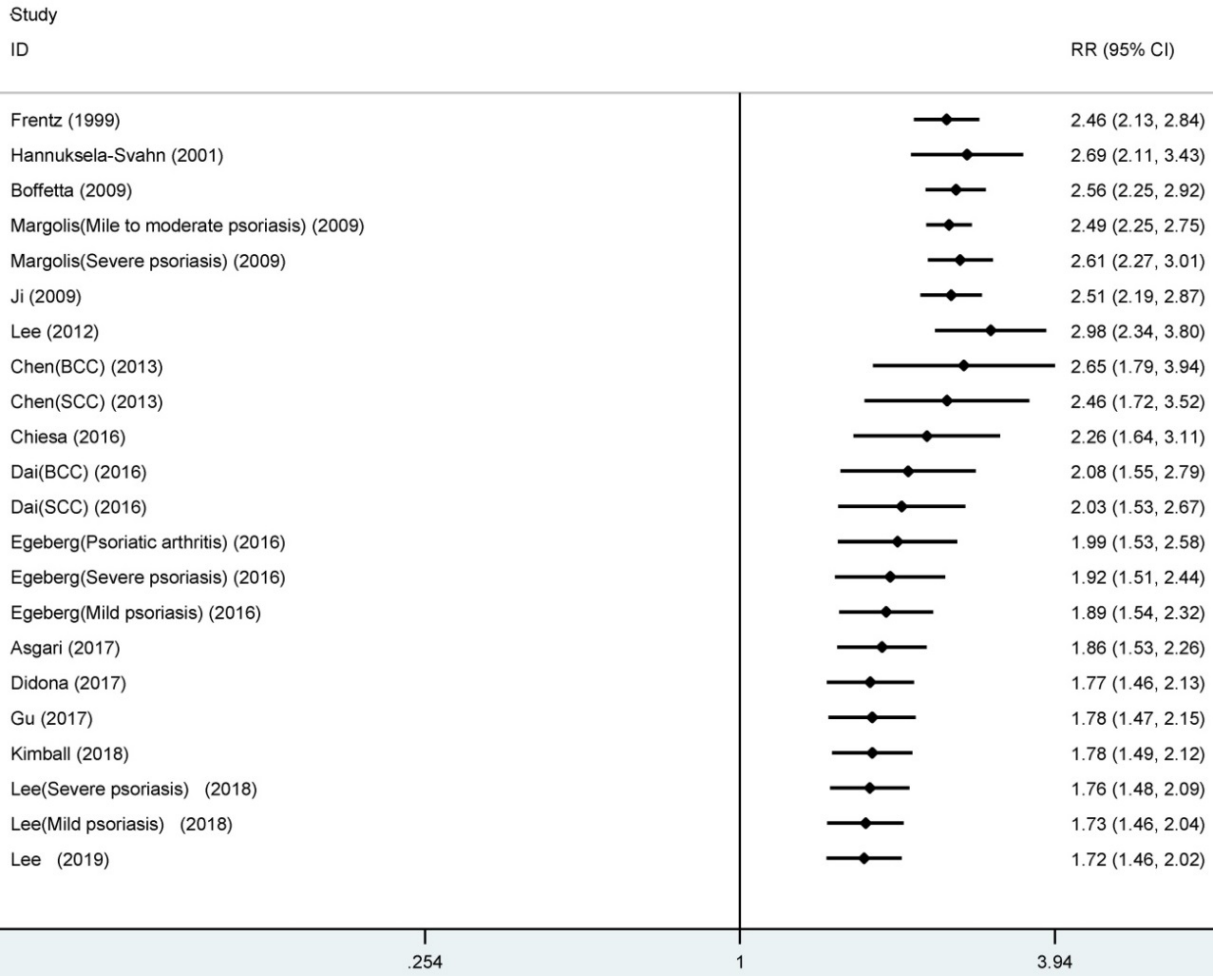
Cumulative influence of study on meta-analysis of association between psoriasis and risk of non-melanoma skin cancer.

Sensitivity analyses were performed by omitting one study at a time and repeating the meta-analysis, showing that the omission of any study does not have a significantly influence on the overall effect (**Figure 4**). We also conducted a further cumulative meta-analysis by calculating the cumulative evidence at the time of each study, which showed that the effect estimates had been consistent over time, providing an indication of the robustness of the results when adding new evidence (**Figure 5**).

## Discussion

### Principal Findings

Current meta-analysis of 16 cohort studies involving over 16,023,503 participants yielded solid evidence that patients with psoriasis could have a higher risk of NMSC than psoriasis-free patients. When stratified by geographic continent, study setting, sex, sample size, follow-up period, study quality, adjustment for major variables, psoriasis severity and pathologic type, the results generally remained constant although there was significant inter-study heterogeneity underlying the pooled results.

Because several common risk factors, such as cigarette smoking, alcohol consumption, obesity, diabetes, and stressful life events, 41-44 have been shown to be shared in patients with psoriasis and cancer, it has been proposed that patients with psoriasis tend to have higher risk of suffering from various diseases, including cancers. Nevertheless, some other studies are also proposed that patients with psoriasis may have a lower risk of cancer. 45 Early hypothesis indicated that psoriasis was a protective factor for skin cancer incidence due to a reduced capacity of psoriatic skin to metabolise precarcinogens caused by impaired arylhydrocarbon hydroxylase (AHH) activity. 46

Previous systematic reviews or meta-analyses have reported the association between psoriasis and the risk of NMSC, which yielded similar findings with the current one. Pouplard et al. summarized 9 studies and showed that patients with psoriasis had an increased risk of NMSC, both for squamous cell carcinoma (RR 5.31, 95% CI 2.63 to 10.71) and basal cell carcinoma (RR 2.00, 95% CI 1.83 to 2.20).^47^ The data by Peleva et al. also suggested that an increased risk of NMSC, particularly squamous cell carcinoma, was reported in patients with psoriasis.^48^ In this updated meta-analysis, we found that psoriasis was also significantly associated with risk of NMSC, but only for squamous cell carcinoma, not for basal cell carcinoma. These findings appeared to be mainly related to the exposure to PUVA treatments, which had reported to have the potential to induce p53 mutations and contribute to development of NMSC in psoriasis patients.^13^

### Strengths and Limitations

The strengths of this updated meta-analysis were presented as follows. First, we thoroughly searched the three major databases without language or publication date limits by applying comprehensive search strategies, making the risk of missing publications less possible. Second, we made rigorously literature screening and eligibility criteria and transparently reported the findings which may minimize the possibility of publication bias. Third, to the best of our knowledge, this meta-analysis included the biggest cohort and most comprehensive analysis with more than 16,023,000 individuals regarding this topic, providing the solidest evidence for the association between psoriasis and risk of NMSC. Fourth, various stratified analyses were performed according to some influential study variables covering the study design, participant characteristics, follow-up period, study quality, exposure and outcome features. The findings were generally in line with the result of the main analysis. Another important strength of our meta-analysis that we should address was that we used a conservative method to combine the risk estimates by using the random effects model. In consequence, the main analysis yielded a considerably high inter-study variance with a prediction interval ranging from 0.81 to 3.89, which indicated that the incidence of NMSC varied considerably in future studies comparing patients with psoriasis to psoriasis-free population. Therefore, considering the variable level of bias from the included observational studies, the significant heterogeneity of the meta-analysis along with the wide prediction interval, the findings of this study from observational studies may cast a shadow upon an uncertainty of the current understanding between psoriasis and risk of NMSC, though significant risk estimate was obtained from the main analysis. From this perspective, our findings of this meta-analysis underline the limitation of observational studies on association of psoriasis with NMSC risk due to a considerable inter-study heterogeneity. We consider that with further advanced methodologies in epidemiological research and RCTs, the potential effects of psoriasis with NMSC risk could be better clarified and stratified based on specific biological or pathophysiologic levels, in which aspects that future studies are warranted.

There are limitations to our study. First, the I^2^ statistic was indicated that considerable inter-study heterogeneity was observed in the meta-analysis, as was expectable since great variations do exist among studies in terms of study design (prospective and retrospective), enrolled subjects, treatment strategy, follow-up period, outcomes combined with some other study features—such heterogeneity could explain partly differences in risk estimates and the associations. Furthermore, we failed to account for exposure and dose of immunosuppressive medications and latent periods for skin cancer due to insufficient data from the original articles included. Second, though some of the original studies have adjusted some major risk factors (age and gender), analysis of association between psoriasis and NMSC risks could have confounded findings. Moreover, these confounding factors remain, including body mass index drinking, smoking status and treatment, and may affect the association of psoriasis with NMSC risk. Therefore, it is strongly suggested that further studies provide detailed individual data regarding the prevalence of associated risk factors in patients with psoriasis and control subjects. Moreover, our study showed a significant variation in terms of measurement of psoriasis and outcome ascertainment among the included studies, such as different diagnostic criteria used for psoriasis and NMSC, different psoriasis treatment regimens and different methodologies to confirm NMSC diagnosis, which should all be uniformed in future studies. Additionally, only three major databases were searched without including others, enhancing the possibilities of some missing studies, though those three databases covered most of available literature. In addition, one conference abstract was included that was not peer reviewed which may lead to potential risk of bias. Finally, grey literature with unpublished data was not searched and we did not contact corresponding authors for the missing original data, which could have led to potential publication bias because studies with positive results might be published more easily than those with negative findings. Though we conducted publication bias analysis by visual inspection of the funnel plot, Egger's regression and Begg's rank correlation tests, no suspicion of small-study effects was indicated. However, we should still interpret the findings with caution.

In summary, the up-to-date evidence from observational studies indicates some extent of association between psoriasis diagnosis and NMSC risk. Due to the large heterogeneity of previously published cohort studies, we propose that in the future, further large prospective cohorts are advocated to validate the association between psoriasis and NMSC risk. Nevertheless, periodic screening for specific cancer risk is warranted in patients with psoriasis.

## Supplementary Material

Supplementary Search Strategy and tables.

## Figures and Tables

**Table 1:** Summary characteristics of included studies.

Author (year)	Country	Study design	Study setting	Cohort sample size	Female (%)	Measure of Psoriasis	Outcome ascertainment	NOS score	Mean/median follow-up period	Adjusted covariates
Lee (2019)	Korea	Prospective cohort	Population-based	1,775,068	38.4	ICD-10	The national cancer registry or hospital admission report	8	15 years	Age, sex, smoking status, alcohol consumption, exercise, body mass index, hypertension and diabetes
Lee (2018)	Korea	Prospective cohort	Population-based	5352534	48.3	ICD-10	ICD-10	8	8 years	Diabetes, hypertension, dyslipidemia, income level and place of residence
Kimball (2018)	USA	Retrospective cohort	Population-based	358132	51.7	ICD-9	ICD-9	8	12 months	NR
Gu (2017)	USA	Retrospective cohort	Population-based	86251	61.2	ICD-9	ICD-9	5	18.6 months	NR
Didona (2017)	Italy	Retrospective cohort	Hospital-based	98695	53.6	ICD-9	ICD-9	4	NR	Gender, age, phototherapyyielded
Asgari (2017)	USA	Retrospective cohort	Population-based	5889	49	ICD-9	ICD-9	7	5.86 years	Presence of psoriatic arthritis, prior ultraviolet light therapy, body mass index, and cigarette use.
Egeberg (2016)	Denmark	Retrospective cohort	Population-based	5559420	50.6	ICD-8 code 173 or ICD-10 code C44	ICD-8 code 173 or ICD-10 code C44	7	16 years	NR
Dai (2016)	USA	Retrospective cohort	Population-based	157934	100	Psoriasis Screening Tool	Psoriasis Screening Tool	7	16 years	Age, BMI, exercise, alcohol intake, smoking, family history of melanoma, nevi counts on extremity,susceptibility to burn, hair color, number of severe or blistering sunburns, and ultraviolet index at birth.
Chiesa (2016)	UK	Retrospective cohort	Population-based	1136082	55.2	Read code	Read code	8	5 years	Age, sex, BMI, drinking and smoking status
Chen (2013)	USA	Mixed retrospective-prospective cohort	Population-based	188406	NR	NR	NR	1	NR	NR
Lee (2012)	China	Retrospective cohort	Population-based	1007061	50.4	ICD-9	ICD-9	7	4.8 years	Age and sex
Ji (2009)	Sweden	Retrospective cohort	Hospital-based	15858	NR	ICD-7,8,9,10	ICD-7	6	10 years	NR
Margolis (2001)	USA	Retrospective cohort	Hospital-based	259808	NR	ICD-9-CM codes	ICD-9-CM codes	6	2.27 years	Age, sex and state of residence
Boffetta (2001)	Sweden	Retrospective cohort	Population-based	9773	45.7	ICD-7,8,9	ICD-7	6	10.6 years	NR
Hannuksela-Svahn (2000)	Finnish	Retrospective cohort	Hospital-based	5687	44.9	The personal identification codes	ICD-9	5	14 years	NR
Frentz (1999)	Danish	Retrospective cohort	Hospital-based	6905	50.5	The personal identification codes	ICD-0	6	9.3 years	NR

Abbreviations: BMI, body mass index; ICD, International Classification of Diseases; NOS, Newcastle-Ottawa Scale; NR, not reported.

**Table 2:** Subgroup analyses for association between psoriasis and risk of non-melanoma skin cancer.

Subgroup	RR	95% CI	I^2^ (%)	No. studies	*P* for interaction
Total	1.72	1.46 to 2.02	96.8	16	-
Geographic continent					<0.001
North America	1.61	1.24 to 2.10	94.5	6	
Europe	1.70	1.33 to 2.16	97.3	7	
Asia	2.01	0.72 to 5.61	95.3	3	
Study setting Population-based Hospital-based	1.63 2.01	1.37 to 1.95 1.14 to 3.53	96.7 97.8	11 5	0.204
Sample size					0.239
<100,000	1.98	1.23 to 3.17	96.7	7	
≥100,000	1.64	1.36 to 1.97	97.1	9	
Follow-up period (Mean/median)					<0.001
<5 years	3.38	2.13 to 5.36	93.7	4	
≥5 years	1.61	1.33 to 1.94	94.8	10	
Adjustment for major variables					<0.001
Yes	1.63	1.26 to 2.12	95.3	8	
No/not reported	1.79	1.52 to 2.11	93.2	8	
Gender					0.828
Male	1.99	0.73 to 5.43	67.4	3	
Female	1.29	1.07 to 1.55	0	2	
NOS score					0.801
High (≥7)	1.56	1.28 to 1.91	97.2	8	
Low to moderate (<7)	1.99	1.39 to 2.84	96.6	8	
Psoriasis severity Mild Moderate to severe Psoriatic arthritis Pathologic type Basal cell carcinoma Squamous cell carcinoma	1.61 1.82 1.62 1.28 2.08	1.25 to 2.09 1.38 to 2.41 1.27 to 2.05 0.81 to 2.00 1.53 to 2.83	96.5 82.7 - 95.8 85.9	7 8 1 4 6	<0.001 <0.001

Abbreviations: CI, confidence interval; RR, relative risk; NOS, Newcastle-Ottawa Scale.

## Abbreviations

CI: confidence interval
HR: hazard ratio
ICD: International Classification of Diseases
MeSH: Medical Subject Headings
MOOSE: the Meta-analysis Of Observational Studies in Epidemiology
NMSC: non-melanoma skin cancer
NOS: the Newcastle-Ottawa scale
OR: odds ratio
PRISMA: the Preferred Reporting Items for Systematic Reviews and Meta-Analyses
PUVA: psoralen plus ultraviolet A
SIR: standardized incidence ratio
