# Supplementary material for: Risk of non-melanoma skin cancer in patients with psoriasis: An updated evidence from systematic review with meta-analysis
Source: J Cancer. 2020 Jan 1;11(5):1047–55. doi: 10.7150/jca.37015 (PMC6959083; doi:10.7150/jca.37015)
Supplement: Supplementary file 1 — Supplementary Search Strategy and tables. [file jcav11p1047s1.pdf]

Supplementary Search Strategy.

Pubmed

1. "Psoriasis"[Mesh]
2. psoriasis[Title]
3. 1 OR 2
4. "Skin Neoplasms"[Mesh]
5. (melanoma OR skin cancer OR cancer OR malignan* OR “keratinocyte carcinoma” OR “basal cell carcinoma” OR “squamous cell carcinoma”)[Title]
6. 4 OR 5
7. 3 AND 6

Embase

1. 'psoriasis'/exp
2. psoriasis:ti
3. 1 OR 2
4. 'skin tumor'/exp
5. (melanoma OR skin cancer OR cancer OR malignan* OR “keratinocyte carcinoma” OR “basal cell carcinoma” OR “squamous cell carcinoma”): ti
6. 4 OR 5
7. 3 AND 6

Cochrane Library Central Register of Controlled Trials

1. MeSH descriptor: [Psoriasis] explode all trees
2. Psoriasis (Word variations have been searched)
3. 1 AND 2
4. MeSH descriptor: [Skin Neoplasms] explode all trees
5. (melanoma OR skin cancer OR cancer OR malignan* OR“keratinocyte carcinoma” OR “basal cell carcinoma” OR “squamous cell carcinoma”):ti (Word variations have been searched)
6. 4 OR 5
7. 3 AND 6

Supplementary Table 1. **The Newcastle–Ottawa scale for selected studies.**

Reference	Selection of exposed cohort	Selection of non-exposed cohort ☆	Ascertainment of exposure ☆	Outcome not present at start ☆	Comparability of cohorts ☆☆	Assessment of outcome ☆	Length of follow-up ☆	Adequacy of follow-up ☆	Total score 9☆
Lee (2019)	☆	☆	☆	☆	☆	☆	☆	☆	8
Lee (2018)	☆	☆	☆	☆	☆	☆	☆	☆	8
Kimball (2018)	☆	☆	☆	☆	☆☆	☆		☆	8
Gu (2017)	☆	☆	☆	☆		☆			5
Didona (2017)	☆		☆	☆		☆			4
Asgari (2017)	☆	☆	☆	☆		☆	☆	☆	7
Egeberg (2016)	☆	☆	☆	☆		☆	☆	☆	7
Dai (2016)		☆	☆		☆☆	☆	☆	☆	7
Chiesa (2016)	☆	☆	☆	☆	☆	☆	☆	☆	8
Chen (2013)		☆							1
Lee (2012)	☆	☆	☆	☆		☆	☆	☆	7
Ji (2009)		☆	☆	☆		☆	☆	☆	6
Margolis (2001)		☆	☆	☆		☆	☆	☆	6
Boffetta (2001)		☆	☆	☆		☆	☆	☆	6
Hannuksela-Svahn (2000)	☆	☆	☆	☆		☆	☆		6
Frentz (1999)		☆	☆	☆		☆	☆	☆	6
